# Prevalence of subthreshold depression and its related factors in Chinese college students: A cross-sectional study

**DOI:** 10.1016/j.heliyon.2024.e32595

**Published:** 2024-06-06

**Authors:** Pu Ge, Cheng Tan, Jia-xin Liu, Qiong Cai, Si-qi Zhao, Wen-ying Hong, Kun-meng Liu, Jia-le Qi, Chen Hu, Wen-li Yu, Yi-miao Li, Yuan You, Jin-han Guo, Ming-yan Hao, Yang Chen, Lu-tong Pan, Di-yue Liu, Meng-yao Yan, Jin-zi Zhang, Qi-yu Li, Bo-ya Sun, Xiao Han, Fuer Mo, Yi-bo Wu, Ying Bian

**Affiliations:** a Institute of Chinese Medical Sciences, University of Macau, Macau, China; b State Key Laboratory of Quality Research in Chinese Medicine, University of Macau, Macau, China; c Department of Public Health and Medicinal Administration, Faculty of Health Sciences, University of Macau, Macau, China; d School of Traditional Chinese Medicine, Beijing University of Chinese Medicine, Beijing, China; e School of Government, Peking University, Beijing, China; f Xiangya School of Nursing, Central South University, Changsha, China; g School of Public Health, Peking University, Beijing, China; h Guangzhou University, Guangzhou, China; i Center for Medical Artificial Intelligence, Qingdao Academy of Chinese Medical Sciences, Shandong University of Traditional Chinese Medicine, Qingdao, China; j School of Journalism&Communication, Zhengzhou University, Zhengzhou, China; k Zijin College of Nanjing University of Technology, Nanjing, China; l School for Sports Humanities and Social Science, Jilin Sport University, Changchun, China; m School of Nursing, Tianjin Medical University, Tianjin, China; n Social, Genetic and Developmental Psychiatry Centre, Institute of Psychiatry, Psychology & Neuroscience, King's College London, London, UK; o Department of Economics, Belarus State University, Minsk, Belarus; p Shanxi Medical University, Shanxi, China; q College of Clinical Chinese Medicine, Hubei University of Chinese Medicine, Wuhan, Hubei Province, China; r School of Public Health, ShanDong University, Jinan, Shandong, China; s International School of Public Health and One Health, Hainan Medical University, Haikou, China; t School of Health Policy and Management, Peking Union Medical College, Chinese Academy of Medical Sciences, Beijing, China; u School of Humanities and Social Sciences, Harbin Medical University, Harbin, China; v School of Humanities and Health Management, Jinzhou Medical University, Jinzhou, China; w Zhejiang University of Media and Communication, Zhejiang, China; x The Fifth Affiliated Hospital of Sun Yat-sat University, Zhuhai, China

**Keywords:** Subthreshold depression, College students, China, Cross-sectional investigation, Mental health, PHQ-9

## Abstract

**Objective:**

To investigate the prevalence of subthreshold depression among Chinese college students and to explore the related factors.

**Methods:**

The research subjects were Chinese college students participating in the “2022 Psychology and Behavior Investigation of Chinese Residents (PBICR-2022)". Data on respondents' general characteristics, quality of life, perceived pressure, family communication, perceived social support, self-efficacy, and depression status were gathered. To investigate the association between each variable and the risk of subthreshold depression, statistical analyses, including chi-square tests and rank sum tests were conducted. Furthermore, a binary stepwise logistic regression was employed to establish the regression model of the factors related to subthreshold depression among Chinese college students.

**Results:**

A prevalence of subthreshold depression of about 39.7 % was found among the 8934 respondents. Logistic regression analysis revealed that respondents who are female, have chronic diseases, are in debt, experience significant impacts from epidemic control policies, have lower self-assessed quality of life, experience challenges in family communication, perceive lower social support, have lower self-efficacy, and feel higher perceived pressure are more likely to develop subthreshold depression compared to the control group. (P < 0.05).

**Conclusion:**

The prevalence rate of subthreshold depression among Chinese college students was found to be approximately 40 %. Female college students suffering from chronic diseases, with households in debt, greatly impacted by epidemic control policies, and experiencing high perceived stress, may be at risk for subthreshold depression among Chinese college students. On the other hand, strong family communication, perceived social support, and self-efficacy were identified as potential protective factors. In order to facilitate timely screening, diagnosis, and treatment of subthreshold depression in Chinese college students, it is crucial for the government, local communities, colleges, and families to prioritize the mental health of college students and implement targeted measures accordingly.

## 1 Background

Subthreshold depression is a state of mental sub-health characterized by depressive symptoms. It refers to a condition in which an individual experiences depressed emotions, loss of interest, or other depressive symptoms but does not meet all the clinical criteria for major depressive disorder (MDD) [1]. It has been demonstrated that it is one of the primary risk factors for Major depressive disorder and may significantly affect people's physical and psychological health [2,3].

Research indicates that subthreshold depression is a prevalent condition worldwide, with a long-standing history. Its prevalence rate in the general population is approximately 20 % throughout the year [4]. Subthreshold depression is a common occurrence among young people [5,6].Previous research has conducted comprehensive surveys on the prevalence of subthreshold depression in young populations. For instance, Langer et al. reported a prevalence rate of 14.3 % among university students in Chile in 2022. [7], and Tan et al. found that the prevalence of subthreshold depression among Chinese university students was 36.56 % in 2011 [8]. According to a study conducted by Yamamoto in 2022 in Japan, the percentage of young adults (aged 18–29) exhibiting symptoms of depression was 20.1 %, which is higher than other age groups [9].

Although subthreshold depression is less persistent and severe than major depressive disorder [10], its high prevalence rate has had a significant impact on the health of young people. Existing studies reveal the impacts of subthreshold depression on mental health, daily life, and self-harm behavior. Research indicates that individuals with subthreshold depression are at a higher risk of developing major depressive disorder [11]. Subthreshold depression can cause significant psychological distress to university students and negatively impact their social activities and academic performance [12,13].It may also hinder the formation of cognitive achievements during work and leisure activities, potentially damaging the quality of life of university students and increasing their risk of suicide [[14], [15], [16]]. Therefore, it is of great practical significance to comprehensively and continuously track the current state and influencing factors of subthreshold depression in university students. This will necessitate timely prevention, detection, diagnosis, and treatment of subthreshold depression.

Current research indicates that subthreshold depression is prevalent among university students and is associated with various factors, including biological aspects, personality traits, university experiences, lifestyle, and environment [17]. Longer sleep duration is associated with a lower likelihood of subthreshold depression [18]. Additionally, outdoor physical exercise can reduce the risk of subthreshold depression [19]. At the same time, positive family relationships, good friendships, and a higher level of perceived social support can reduce the risk of subthreshold depression [[20], [21], [22]]. The COVID-19 pandemic has had a negative impact on the mental health of university students worldwide. This may have led to an increase in the prevalence of subthreshold depression, as pandemic prevention and isolation measures have reduced students' social contact and support. Additionally, the financial difficulties and potential economic losses during the pandemic could cause significant stress and psychological distress for university students [[23], [24], [25], [26], [27], [28]]. Currently, there is a dearth of extensive, national-level research on the factors associated with subthreshold depression among university students in China.

This study aims to determine the prevalence of subthreshold depression among Chinese college students and investigate its associations with general characteristics, self-assessment quality of life, perceived pressure, family communication, perceived social support, and self-efficacy. The aim of this research is to raise awareness and attention towards the mental health of Chinese college students. We aim to identify the risk factors of subthreshold depression and provide early diagnosis, treatment, and identification to alleviate the impact of subthreshold depression on this vulnerable population and promote their overall mental well-being.

## 2 Methods

### 2.1 Study design

The study data was from the 2022 Psychology and Behavior Investigation of Chinese Residents (PBICR-2022). From June 20 to August 31, 2022, the investigation was carried out. Based on the findings of the 7th Census of China, which was conducted in 2021, a multi-stage sample of Chinese citizens living in 31 provincial administrations, 148 cities, 202 districts and counties, 390 townships/towns/streets, and 780 communities/villages were investigated. (Hong Kong, Macau, and Taiwan were not included in the investigation locations.) A total of 31,480 questionnaires were given out to the public by trained investigators on a one-to-one, face-to-face basis, and 30,505 valid questionnaires were collected, representing a 96.9 % effective return rate. PBICR-2022 has been officially registered in the Chinese Clinical Trials Registry (registration number: ChiCTR2200061046). The applicable criteria and study plan were rigorously followed during the study execution procedure. The study population involved in this paper was Chinese college students enrolled in PBICR-2022. This study was approved by the Ethics Research Committee of the Health Culture Research Center of Shaanxi (No. JKWH-2022-02). All research procedures were conducted by the principles outlined in the Declaration of Helsinki and the guidelines provided by the relevant regulatory bodies. The research was conducted with the voluntary participation of human subjects who provided written informed consent before being included in the study. Participant confidentiality and anonymity were preserved throughout the data collection, analysis, and reporting processes. Measures were taken to ensure that no harm came to any participants as a result of their involvement in the study. Any potential risks or adverse effects of the research were carefully considered and managed to minimize harm. Data was stored securely and accessed only by authorized personnel for purposes related to the research project. Results have been reported in aggregate form and individual participants cannot be identified from the presented data.

## 3 Participants

### 3.1 Calculation of the minimum sample size

**Fig. 1:**
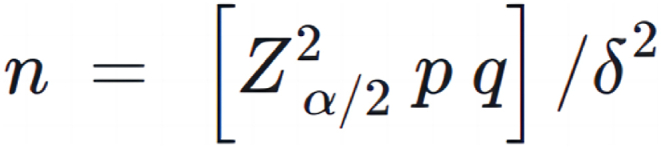
Formula for calculating the minimum sample size of this study.

The minimum sample size for the study was calculated by the formula in Fig. 1 [29].

In the formula, n represents the sample size, p represents the estimated prevalence of subthreshold depression among Chinese college students, q = 1-p, α = 0.05, Zα/2 = 1.96 ≈ 2, and δ is the acceptable error, δ = 0.1*p. Referring to the pertinent literature, the prevalence rate of subthreshold depression among college students ranged from 14 % to 39 % [12,30], and based on the above prevalence, the minimum sample size was calculated to be 2458. Considering that there might be 20 % of invalid questionnaires, the minimum sample size obtained by further calculation was 3073.

### 3.2 Inclusion criteria

① Must be at least 18 years old and no older than 30 years old.
②Have Chinese nationality.
③Students enrolled in Chinese colleges, including graduate, undergraduate, and tertiary students.
④Volunteer to participate in this study and fill in the informed consent form.
⑤Complete the questionnaire by themselves or with the assistance of the investigator.

### 3.3 Exclusion criteria

①Suffered from moderate or severe depression (score of the Patient Health Questionnaire (PHQ-9) was bigger than or equal to 10). This group was not excluded when calculating the prevalence of subthreshold depression among college students, and was not analyzed when conducting the analysis of factors associated with the risk of developing subthreshold depression.
②Recently have participated in other similar studies.
③Limited in mobility, unconscious or psychopathic.
④Those whose filled-in college name does not match with the study section, e.g. the filled-in college is a Vocational and Technical College (postsecondary specialised college), but the filled-in study section is PhD.
⑤Those whose information filled in is incomplete, such as the specific name of the university attended was not filled in.

**Fig. 2:**
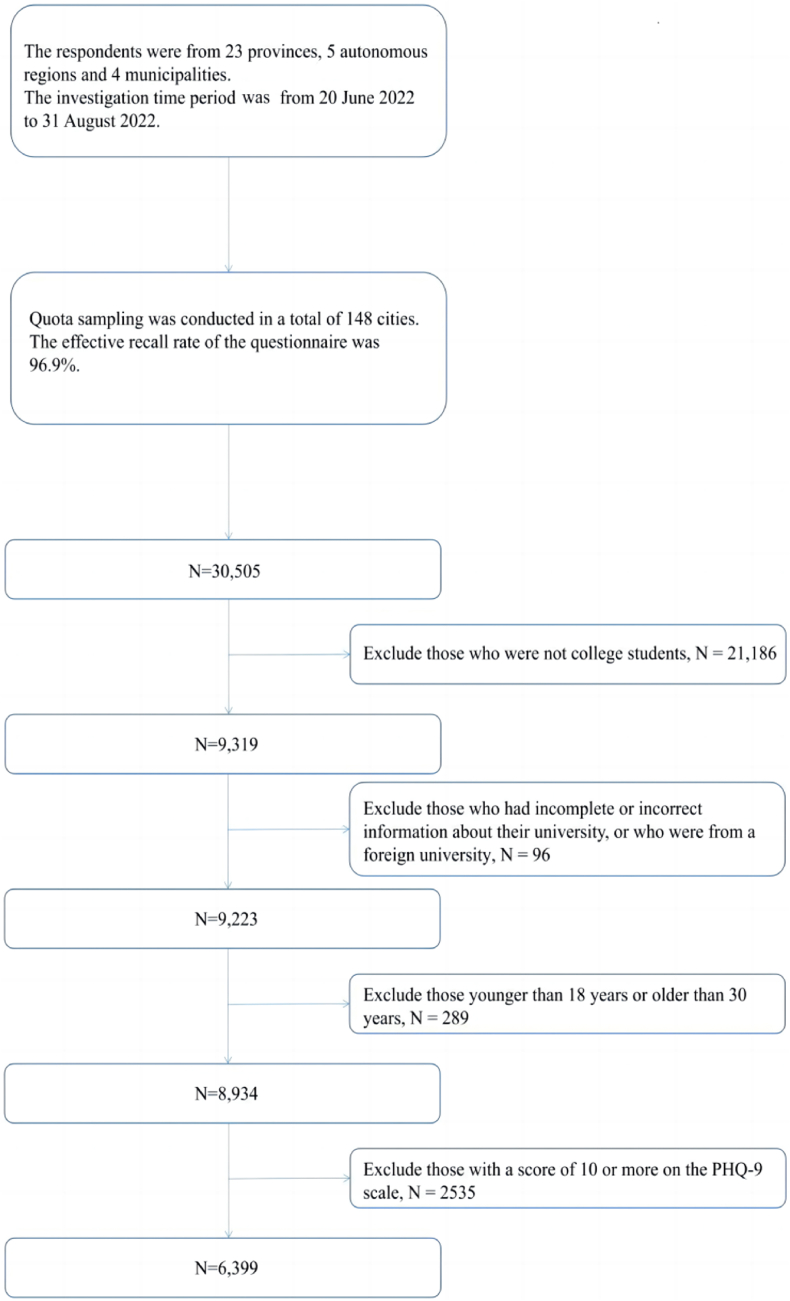
Flowchart of participant enrollment.

The inclusion and exclusion of the study subjects is shown in Fig. 2.

## 4 Research instruments

The research instruments utilized in this study were divided into 3 elements. The first part investigated the general characteristics of the respondents. The second part investigated the depression status of the respondents by the Patient Health Questionnaire-9 (PHQ-9). The third part is a series of other standard scales, including the EuroQol Five Dimensions Questionnaire Visual Analogue Scale (EQ-5D-VAS), the Perceived Stress Scale-4 items (PSS-4), the Family Communication Scale (FCS-10), the Perceived Social Support Scale Short Form-3 items (PSSS-SF3), and the New General Self-efficacy Scale Short Form-3 items (NGSES-SF3). The instruments were used to investigate respondents' quality of life, perceived stress, family communication status, perceived social support status, and self-efficacy levels.

### 4.1 General characteristics of respondents

The first part of the questionnaire investigated the general characteristics of the respondents, including gender, age, ethnicity, college or university, current academic section, major, whether they had chronic diseases, province, current residence (urban or rural), whether they were only-children, whether their families were in debt, per capita monthly household income, and whether the epidemic prevention and control policies had a significant impact on their lives. The questions on age and university were filled in the blanks, and the question on the impact of the epidemic prevention and control policy on the respondents' life was measured by the visual simulation scoring method (a scale from 0 to 100 was attached to the question, with 0 indicating that the epidemic prevention and control policy had no impact on the respondents and 100 indicating that the epidemic prevention and control policy had a great impact on the respondents, and the respondents chose the appropriate number to meet their situation by sliding the swim code on the scale). The other questions were all single-choice questions. In the question of the impact of epidemic prevention and control policies on respondents' lives, respondents' scores did not conform to a normal distribution, and the median score was 62. Respondents who scored 62 or less in this question were categorized into the “little impact of epidemic prevention and control policies on their lives" group, and those who scored 63 or more were categorized into the “significant impact of epidemic prevention and control policies on their lives" group.

### 4.2 PHQ-9

The depression status of the respondents was assessed by the PHQ-9, whose reliability and validity have been validated in several previous studies. As a depression self-assessment instrument, the scale is widely used internationally because it can quickly and effectively screen one's depression status. The scale consists of nine items that participants rate according to their feelings in the past two weeks, and scores on a four-point scale (0 = Not at All; 3 = Nearly every day; the scale scores range from 0 to 27, with higher scores indicating a more severe depressive condition in the respondent). In this study, the Cronbach's α for the PHQ-9 was 0.796, with a split-half reliability of 0.700, which is a relatively good reliability. The PHQ-9 scale had a KMO value of 0.94, and the p-value of Bartlett's test of sphericity was less than 0.001. Validated factor analysis indicated that each item of the PHQ-9 scale had a standardized factor loading above 0.639. The AVE value of the PHQ-9 scale was 0.548, and the CR value was 0.915, indicating good convergent validity and construct reliability. The fitness indexes for the scale model are as follows: GFI = 0.954 (>0.9),RMR = 0.023 (<0.05), CFI = 0.954 (>0.9), and NFI = 0.954 (>0.9). These results suggest that the PHQ-9 scale has good construct validity in this study. With reference to the Pertinent literature [31,32], respondents scoring 0–4 on this scale were categorized as not depressed, and those scoring 5–9 were categorized as patients with subthreshold depression, and those scoring 10 or above were categorized as patients with depression. For the analysis of things associated with the risk of subthreshold depression, solely respondents with many 0–9 on this scale were analyzed.

### 4.3 Other scales

#### 4.3.1 EQ-5D-VAS

Respondents' self-rated quality of life was assessed by the EQ-5D-VAS, which is the most commonly used instrument to measure Health-Related Quality of life (HRQoL) as well as assess the mental health and sanity of respondents. The EuroQol Five Dimensions Questionnaire Visual Analogue Scale (EQ-5D-VAS) is part of the EQ-5D-5L and is used for subjective assessment of health-related quality of life of respondents. In 2015, Hong et al. analyzed the factors associated with the quality of life among Korean COPD patients by EQ-5D-VAS. In previous studies, the EQ-5D-VAS has shown good metrological properties. It investigates participants' self-rated health status by using numbers between 0 and 100, with 100 representing the best health status and 0 representing the worst health status [33]. The EQ-5D-VAS consists of only 1 item, so its reliability was not assessed in this study. Referring to the Pertinent literature, the respondents were divided into two groups according to their self-rated quality of life based on respective EQ-5D-VAS scores: the high group (81–100) and the low group (80 and below [34]).

#### 4.3.2 Perceived Stress Scale-4

The Perceived Stress Scale was used to assess the respondents' perceived stress levels. The Perceived Stress Scale can be used to measure an individual's perceived level of psychological stress which is divided into two dimensions: sense of loss of control and sense of tension. The Scale consists of four entries on a five-point scale (0 = never; 4 = always; scale scores range from 0 to 16, with higher scores indicating more severe perceived stress by the respondent). Cohen's study demonstrated that PSS-4 has good reliability and validity [35]. The split-half reliability of the PSS-4 in this study was 0.769, which is a relatively good reliability. Since the Corresponding scores on this scale did not conform to a normal distribution, the respondents were divided into two groups according to the median score (6) on the Perceived Stress Scale: the high group (7 and above) and the low group (6 and below).

#### 4.3.3 Family Communication Scale

The Family Communication Scale was used to evaluate the respondents' family communication. The Family Communication Scale contains 10 entries on a five-point Likert scale (1 = strongly disagree; 5 = strongly agree; scale scores range from 10 to 50, with higher scores indicating better family communication of the respondent [36]). The purpose of the scale is to measure the quality of communication between family members, which involves the exchange of ideas, The exchange of information, degree of concern, degree of openness, confidence and emotion between family members. The Cronbach's α of the FCS-10 in this study was 0.972, and the split-half reliability was 0.960, which indicated good reliability in the study. The FCS-10 scale had a KMO value of 0.966, and the p-value of Bartlett's test of sphericity was less than 0.001. Validated factor analysis indicated that each item of the FCS-10 scale had a standardized factor loading above 0.812. The FCS-10 scale had good convergent validity and construct reliability, as evidenced by an AVE value of 0.779 and a CR value of 0.972. The scale model fitness indicators are as follows:

GFI = 0.959 (>0.9),RMR = 0.022 (<0.05),CFI = 0.959 (>0.9),NFI = 0.959 (>0.9). The above results indicate that the construct validity of the FCS-10 scale is good in this study. Since the respondents' scores in this scale did not conform to a normal distribution, the respondents were divided into two groups based on the median score (39) of their Family Communication Scale scores: high group (40 and above) and low group (39 and below).

#### 4.3.4 Perceived social support Scale Short Form

Respondents' perception of social support was measured by the Perceived Social Support Scale Short Form, which contains three items on a seven-point Likert scale (1 = strongly disagree; 7 = strongly agree; scale scores range from 3 to 21, with higher scores indicating higher levels of perceived social support [37]). The Perceived Social Support Scale short form is a simplified version of the Perceived Social Support Scale (PSSS-12), which was developed by members of PBICR-2022, and is a social support scale that emphasizes self-understanding and self-perception [38]. It measures respondents' perception level of support from various sources of social support, including three dimensions: family support, friend support, and other people's support. The Cronbach's α for PSSS-3 in this study was 0.902, and the split-half reliability was 0.799, with good reliability. The PSSS-3 scale had a KMO value of 0.751 and a Bartlett's test of sphericity p-value of less than 0.001. Validated factor analysis indicated that each entry of the PSSS-3 scale had a standardized factor loading above 0.852. The scale had good convergent validity and construct reliability, with an AVE value of 0.755 and a CR value of 0.903. Since the respondents' scores in this scale did not conform to a normal distribution, the respondents were divided into two groups according to the median score (16) of their PSSS-3 scores: the high group (17 and above) and the low group (16 and below).

#### 4.3.5 New General Self-efficacy Scale Short Form

Respondents' self-efficacy was measured by the New General Self-Efficacy Scale Short Form, which includes three entries on a five-point Likert scale (1 = strongly disagree; 5 = strongly agree; scale scores range from 3 to 15, with higher scores indicating higher levels of respondents' self-efficacy). The New General Self-Efficacy Scale Short Form (NGSES-SF3) [39] was simplified from the New General Self-Efficacy Scale (NGSES-8) by members of PBICR-2022, and includes three items: whether they can perform difficult tasks, whether they can successfully overcome many challenges, and whether they have the confidence to perform many different tasks effectively. The Cronbach's α of the NGSES-SF3 in this study was 0.951, and the split-half reliability was 0.852, which indicates good reliability. The KMO value for the NGSES-SF3 scale was 0.774, with a Bartlett's test of sphericity p-value of less than 0.001. Validated factor analysis showed that the standardized factor loadings for each entry of the NGSES-SF3 scale were above 0.913. The AVE value of the NGSES-SF3 scale was 0.868, and the CR value was 0.952, indicating good convergent validity and construct reliability. Since the respondents' scores in this scale did not conform to a normal distribution, the respondents were divided into two groups according to their self-efficacy status: high group (13 and above) and low group (12 and below) based on the median score (12) of the respondents' scores on the NGSES-SF3.

## 5 Data analysis

Data entry and analysis were performed by SPSS™ for Windows (version 25.0). The scale scores were transformed into dichotomous variables (high and low subgroups) based on the relevant literature. Categorical variables were expressed as composition ratios.T-tests were used to compare differences in each item of PHQ-9 scale scores between subthreshold depressed and non-subthreshold depressed respondents. Chi-square tests and rank sum tests were used to perform between-group comparisons of the prevalence of subthreshold depression. The study conducted a multifactorial analysis of the factors associated with the risk of subthreshold depression among the respondents using multivariate binary stepwise logistic regression. The inclusion and exclusion criteria for the variables were *P* = 0.05 and *P* = 0.10, respectively. The study examined the general characteristics of the respondents and their rating on various scales, including EQ-5D-VAS, PSS, FCS-10, PSSS-3, and NGSES-SF3, as independent variables. The dependent variable was the presence of subthreshold depression. To ensure reliable results, the study evaluated the internal consistency reliability of the scales used and investigated the presence of common method bias using the Harman one-way method. Furthermore, the respondents were categorized into subgroups based on gender, profession, and location. Logistic regression models were constructed separately to examine the variations in factors linked to the risk of subthreshold depression among different subgroups of the population. Unless otherwise stated, the level of statistical tests was α = 0.05, and all tests were two-sided.

## 6 Quality control

PBICR-2022 conducted quality control in five main stages: questionnaire design, pilot study, investigator training, questionnaire distribution and questionnaire screening. See Supplementary Table 1 for details.

## 7 Result

### 7.1 Common method variance test

The study employed Harman's single factor method to test for common method bias. Therefore, there is no significant common method bias. The results revealed six factors with characteristic roots greater than one. The first main factor had a variance contribution rate of 34.8 %, which is less than the threshold of 40 %.

### 7.2 General characteristics and subthreshold depressive illness of respondents

**Fig. 3:**
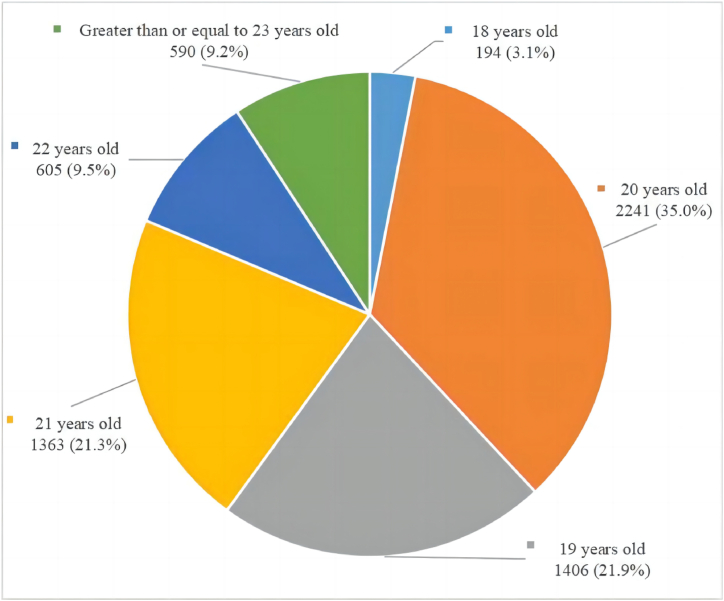
Age distribution of respondents.

After screening, 8934 respondents were surveyed for this study. Among them, 2535 respondents scored 10 points or more in the PHQ-9 and may be suffering from depression, resulting in a prevalence rate of approximately 28.4 %. Additionally, 3550 respondents scored between 5 and 9 points in the PHQ-9 and may have subthreshold depression, resulting in a prevalence rate of approximately 39.7 %. This study analyzed 6399 non-depressed respondents, of which 55.5 % had subthreshold depression. Table 1 and Fig. 3 describe the general characteristics of the respondents. Of the 6399 respondents, 60.9 % (3896) were female, 85.6 % (5478) were undergraduate students, and 58.3 % (3732) majored in non-social science subjects. Regarding age, the majority of respondents fell within the 19–22 age range (5615 respondents, 87.7 %, as shown in Fig. 3). Additional general characteristics of the respondents are presented in Table 1.

**Table 1:** General characteristics, scale score grading and chi-square test results of the respondents.

Categorical variables	Number and percentage of people	Whether the respondent suffers from subthreshold depression		χ2	P-values
		Number and percentage of people without subthreshold depression	Number and percentage of people with subthreshold depression		
Total people	6399 (100.0 %)	2849 (44.5 %)	3550 (55.5 %)	–	–
Gender				29.625	**<0.001**
Male	2503 (39.1 %)	1220 (48.7 %)	1283 (51.3 %)		
Female	3896 (60.9 %)	1629 (41.8 %)	2267 (58.2 %)		
Ethnicity				0.637	0.425
Han	5703 (89.1 %)	2549 (44.7 %)	3154 (55.3 %)		
Ethnic Minorities	696 (10.9 %)	300 (43.1 %)	396 (56.9 %)		
Currently enrolled in school period				0.461	0.794
Junior College	639 (10.0 %)	277 (43.3 %)	362 (56.7 %)		
Undergraduate	5478 (85.6 %)	2444 (44.6 %)	3034 (55.4 %)		
Postgraduate	282 (4.4 %)	128 (45.4 %)	154 (54.6 %)		
Major				14.030	**<0.001**
Social Science	2667 (41.7 %)	1114 (41.8 %)	1553 (58.2 %)		
Non-social Science	3732 (58.3 %)	1735 (46.5 %)	1997 (53.5 %)		
Whether suffering from chronic diseases				34.681	**<0.001**
No	5865 (91.7 %)	2676 (45.6 %)	3189 (54.4 %)		
Yes	534 (8.3 %)	173 (32.4 %)	361 (67.6 %)		
Location				8.013	**0.018**
Western China	2026 (31.7 %)	872 (43.0 %)	1154 (57.0 %)		
Middle China	1716 (26.8 %)	813 (47.4 %)	903 (52.6 %)		
Eastern China	2657 (41.5 %)	1164 (43.8 %)	1493 (56.2 %)		
Place of permanent residence				0.698	**0.403**
Rural	1410 (22.0 %)	614 (43.5 %)	796 (56.5 %)		
Urban	4989 (78.0 %)	2235 (44.8 %)	2754 (55.2 %)		
Whether is an only child				13.617	**<0.001**
No	3876 (60.6 %)	1654 (42.7 %)	2222 (57.3 %)		
Yes	2523 (39.4 %)	1195 (47.4 %)	1328 (52.6 %)		
Categorical variables	Number and percentage of people	Whether the respondent suffers from subthreshold depression		χ2	P-values
		Number and percentage of people without subthreshold depression	Number and percentage of people with subthreshold depression		
Whether the respondent's household is in debt				35.117	**<0.001**
No	3703 (57.9 %)	1765 (47.7 %)	1938 (52.3 %)		
Yes	2696 (42.1 %)	1084 (40.2 %)	1612 (59.8 %)		
Monthly per capita household income (Unit:RMB)				10.155	**0.001**
3000 ($432.9004) or below	2124 (33.2 %)	886 (41.7 %)	1238 (58.3 %)		
3001 ($433.04473) or above	4275 (66.8 %)	1963 (45.9 %)	2312 (54.1 %)		
Whether the epidemic prevention and control policy have a great impact on respondents				13.873	**<0.001**
No	3223 (50.4 %)	1509 (46.8 %)	1714 (53.2 %)		
Yes	3176 (49.6 %)	1340 (42.2 %)	1836 (57.8 %)		
Self-assessment of quality of life				240.205	**<0.001**
Low score group	2995 (46.8 %)	1026 (34.3 %)	1969 (65.7 %)		
High score group	3404 (53.2 %)	1823 (53.6 %)	1581 (46.4 %)		
Perceived pressure				232.506	**<0.001**
Low score group	3532 (55.2 %)	1874 (53.1 %)	1658 (46.9 %)		
High score group	2867 (44.8 %)	975 (34.0 %)	1892 (66.0 %)		
Family Communication				211.657	**<0.001**
Low score group	3275 (51.2 %)	1169 (35.7 %)	2106 (64.3 %)		
High score group	3124 (48.8 %)	1680 (53.8 %)	1444 (46.2 %)		
Perceived Social Support				330.771	**<0.001**
Low score group	3543 (55.4 %)	1218 (34.4 %)	2325 (65.6 %)		
High score group	2856 (44.6 %)	1631 (57.1 %)	1225 (42.9 %)		
Self efficacy				205.781	**<0.001**
Low score group	5549 (86.7 %)	2277 (41.0 %)	3272 (59.0 %)		
High score group	850 (13.3 %)	572 (67.3 %)	278 (32.7 %)

Of the 6399 respondents included in univariate and multivariate analyses, the total scores on the PHQ-9 were found to be non-normally distributed (Kolmogorov-Smirnov test *P* < 0.001). The score had a mean of 4.83 ± 3.108 and a median of 5 points, with lower and upper quartiles of 2 and 8 points respectively. Referring to Pertinent literature [40,41], the respondents were divided into non-subthreshold depression respondents (2849, 44.5 %) and subthreshold depression respondents (3550, 55.5 %) with a 4-point cut-off value.

### 7.3 Scores of each item on the PHQ-9 in respondents with subthreshold depression and non-subthreshold depression

**Fig. 4:**
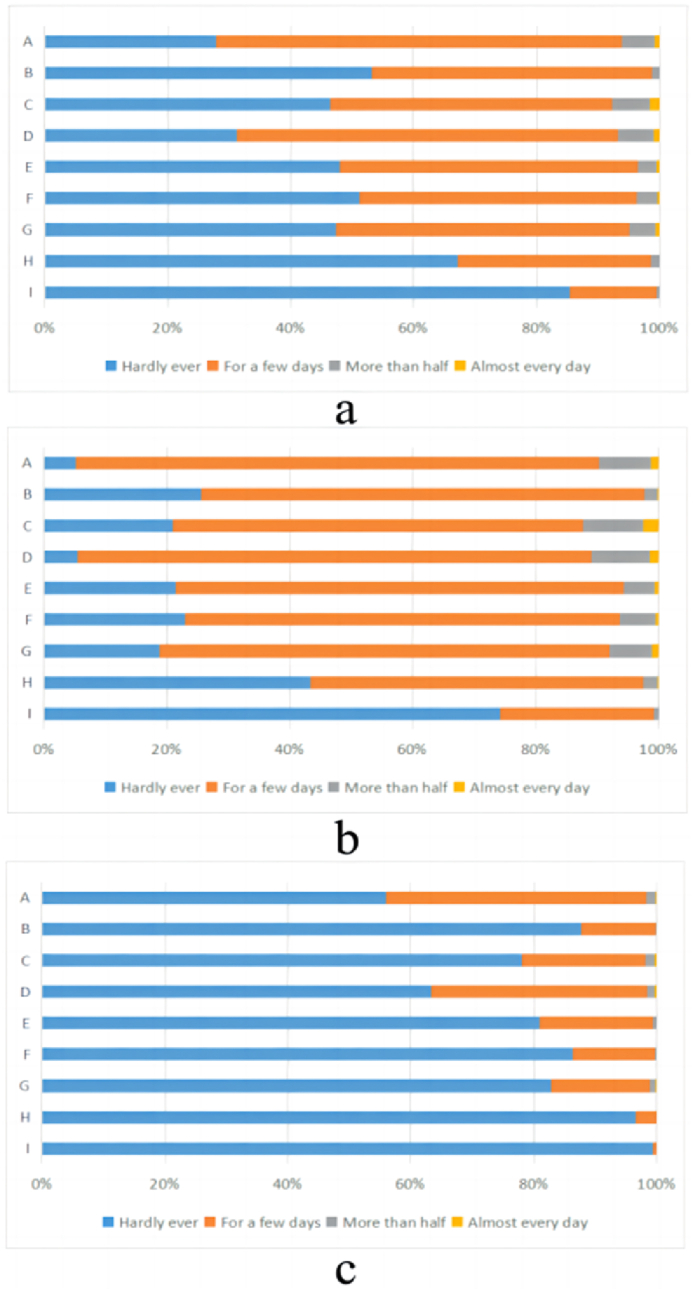
Respondents' scores on each item of PHQ-9. (a: all respondents; b: subthreshold depressed respondents; c: non-subthreshold depressed respondents).

T-test was used to compare the differences in each item of PHQ-9 between subthreshold depressed and non-subthreshold depressed respondents. The results of T-test showed that there were significant differences in the mean values of each item of PHQ-9 between the two groups (*P* < 0.05). The largest differences in mean scores between the two groups were found in item G (0.718 95%CI 0.694, 0.741) and item F (0.698 95%CI 0.676, 0.719) (G: Trouble concentrating on things, such as reading the newspaper or watching television; F: Feeling bad about yourself - or that you are a failure or have let yourself or your family down). While the two groups had the smallest mean differences in scores for item I (0.258 95%CI 0.243, 0.274) and H (0.557 95%CI 0.538, 0.577) (I: Thoughts that you would be better off dead or of hurting yourself in some way; H: Moving or speaking so slowly that other people could have noticed? Or the opposite—being so fidgety or restless that you have been moving around a lot more than usual, or the opposite—being so fidgety or restless that you have been moving around a lot more than usual). The specific results are shown in Table 2 and Fig. 4(a–c).

**Table 2:** Scores and *t*-test results for each item on the PHQ-9 for subthreshold and non-subthreshold depressed individuals.

Item	Group	Mean ± SD	95%CI of Mean	Mean difference	95%CI of Mean difference	t	*P*
A	Non-subthreshold depression	0.46 ± 0.534	(0.44, 0.48)	0.600	(0.576, 0.624)	48.730	＜**0.001**
	subthreshold depression	1.06 ± 0.427	(1.04, 1.07)				
B	Non-subthreshold depression	0.12 ± 0.334	(0.11, 0.14)	0.644	(0.624, 0.664)	63.204	＜**0.001**
	subthreshold depression	0.77 ± 0.479	(0.75, 0.78)				
C	Non-subthreshold depression	0.24 ± 0.482	(0.22, 0.26)	0.696	(0.668, 0.724)	48.292	＜**0.001**
	subthreshold depression	0.94 ± 0.636	(0.92, 0.96)				
D	Non-subthreshold depression	0.39 ± 0.529	(0.37, 0.41)	0.682	(0.658, 0.707)	54.725	＜**0.001**
	subthreshold depression	1.07 ± 0.451	(1.05, 1.08)				
E	Non-subthreshold depression	0.20 ± 0.415	(0.18, 0.21)	0.653	(0.630, 0.676)	55.886	＜**0.001**
	subthreshold depression	0.85 ± 0.520	(0.83, 0.87)				
F	Non-subthreshold depression	0.14 ± 0.360	(0.13, 0.15)	0.698	(0.676, 0.719)	62.539	＜**0.001**
	subthreshold depression	0.84 ± 0.530	(0.82, 0.86)				
G	Non-subthreshold depression	0.18 ± 0.422	(0.17, 0.20)	0.718	(0.694, 0.741)	59.705	＜**0.001**
	subthreshold depression	0.90 ± 0.540	(0.88, 0.92)				
H	Non-subthreshold depression	0.03 ± 0.186	(0.03, 0.04)	0.557	(0.538, 0.577)	56.958	＜**0.001**
	subthreshold depression	0.59 ± 0.545	(0.57, 0.61)				
I	Non-subthreshold depression	0.01 ± 0.090	(0.00, 0.01)	0.258	(0.243, 0.274)	32.657	＜**0.001**
	subthreshold depression	0.27 ± 0.460	(0.25, 0.28)				

Note.

A:Little interest or pleasure in doing things.

B:Feeling down, depressed, or hopeless.

C:Trouble falling or staying asleep, or sleeping too much.

D:Feeling tired or having little energy.

E:Poor appetite or overeating.

F:Feeling bad about yourself—or that you are a failure or have let yourself or your family down.

G:Trouble concentrating on things, such as reading the newspaper or watching television.

H:Moving or speaking so slowly that other people could have noticed? Or the opposite—being so fidgety or restless that you have been moving around a lot more than usual.

I:Thoughts that you would be better off dead or of hurting yourself in some way.

### 7.4 The respondents' scores on other scales

The respondents' scores on other scales are shown in Table 3. The scores of all scales did not conform to normal distribution (Kolmogorov-Smirnov test *P* < 0.001).

**Table 3:** Scores on the scales used in the study.

Scale	Variable measured	Number of items	Score range	Mean ± SD	Median (Lower quartile, upper quartile)	Truncation value	Number and percentage of low score group	Number and percentage of high score group
PHQ-9	Depression status	9	0–27	4.83 ± 3.108	5 (2, 8)	4	2849 (44.5 %)a	3550 (55.5 %)b
PSS-4	Perceived stress	4	0–16	5.41 ± 2.798	6 (4, 7)	6	3939 (61.6 %)	2460 (38.4 %)
FCS-10	Family communication	10	10–50	37.60 ± 8.638	39 (31, 43)	39	3275 (51.2 %)	3124 (48.8 %)
PSSS-SF3	Perceived social support	3	3–21	10.40 ± 2.358	11 (9, 12)	11	3532 (55.2 %)	2867 (44.8 %)
NGSES-SF3	Self efficacy	3	3–15	10.83 ± 2.398	12 (9, 12)	12	5549 (86.7 %)	850 (13.3 %)
EQ-5D-VAS	Self-assessment of quality of life	1	0–100	77.46 ± 20.988	81 (70, 91)	80	2995 (46.8 %)	3404 (53.2 %)

a: number and percentage of people without subthreshold depression.

b: number and percentage of people with subthreshold depression.

### 7.5 Univariate analysis of subthreshold depression related factors

The rank sum test (Mann-Whitney *U* test) was used to analyze the difference in age distribution between the subthreshold and non-subthreshold depressed respondents. The results of rank sum test showed that the mean rank of age of respondents without subthreshold depression was 3182.73, the mean rank of age of respondents with subthreshold depression was 3213.86, the standardized test statistic was −0.692, and the *P*-value of rank sum test was 0.489, indicating that there was no significant difference in age distribution between subthreshold depressed and non-subthreshold depressed respondents.

**Fig. 5:**
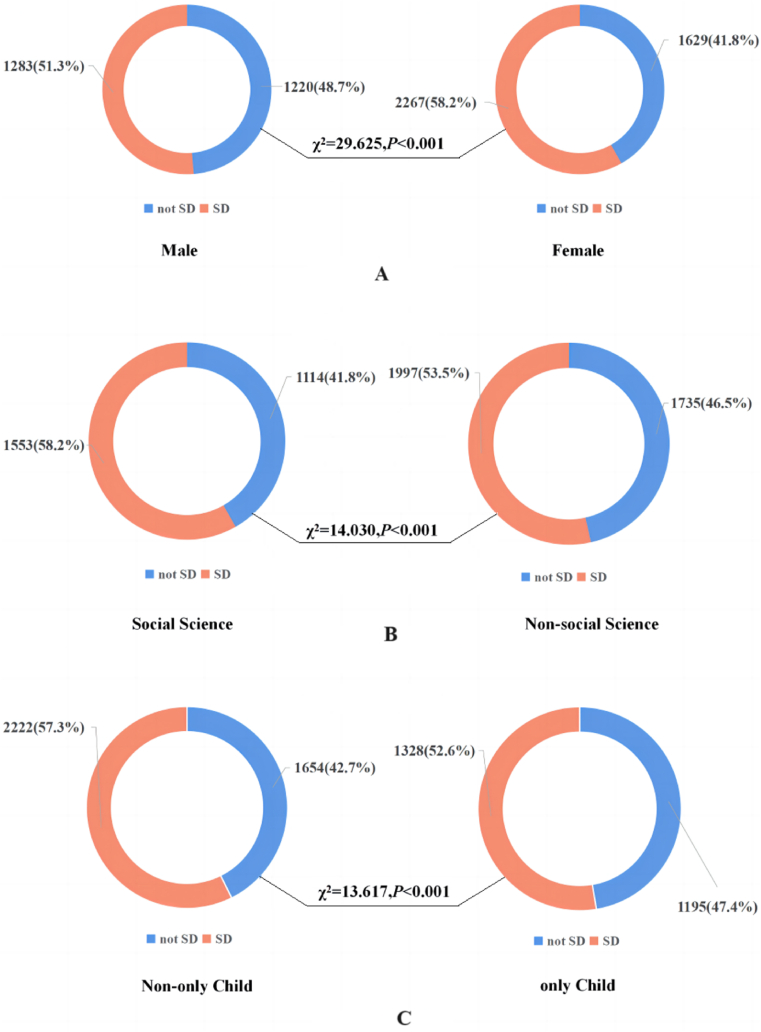
Prevalence of subthreshold depression among respondents by gender (Fig. 5(A)), major (Fig. 5(B)) and only-child status (Fig. 5(C)). Note: SD: Subthreshold Depression.

Chi-square test was used to analyze the correlation between other factors and the risk of subthreshold depression. The results showed that there were significant differences in the risk of subthreshold depression among respondents of different gender, major, chronic disease status, location, only-child status, family debt status, per capita monthly family income, impact of epidemic control policies on life, self-rated quality of life, perceived pressure, family communication status, perceived social support and self-efficacy (*P* < 0.05) (see Fig. 5 and Table 3).

### 7.6 Binary logistic regression results of subthreshold depression related factors

**Fig. 6:**
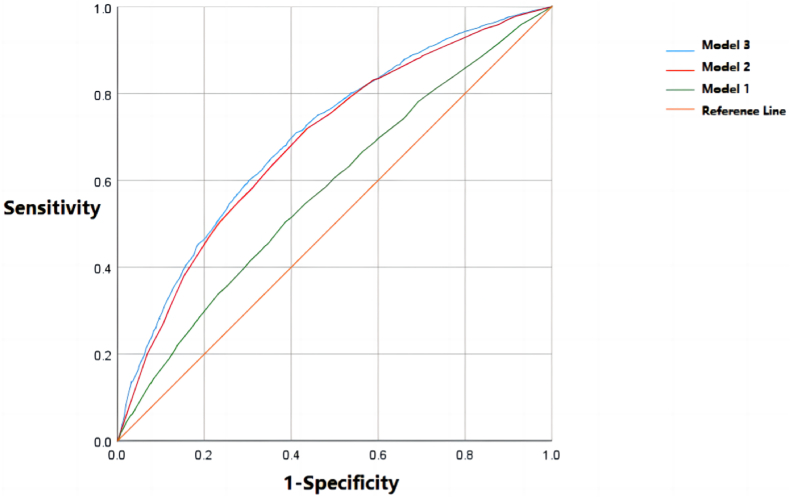
ROC curve for logistic regression models.

The multivariate analysis of the factors related to the risk of subthreshold depression in Chinese university students was carried out by using multi-factor step-by-step binary logistic regression. In each regression model, the dependent variable was whether the respondents had subthreshold depression. The samples used for model establishment were the 6399 respondents with PHQ-9 scores equal to or below 9 points. In order to ensure the robustness of the model, a total of three models were established. Model 1 took the general characteristics of the respondents as the independent variables; Model 2 took the scale score grading of the respondents as the independent variables; Model 3 took the general characteristics and scale score grading of the respondents as the independent variables, and Model 3 is the main result of this study. Meanwhile, the ROC curves of the three models were drawn (see Fig. 6) to evaluate the quality of the models. According to the ROC curves, the quality of Model 3 is better than that of Models 1 and 2. The classification and evaluation indexes of each model are shown in Table 4. The accuracy rate, recall rate, Yoden index and other indexes also showed that the quality of Model 3 is higher than that of Model 1 and Model 2. Among them, the AUC value of the ROC curve of Model 3 is 0.701, which is greater than 0.7, indicating that its quality is acceptable, while the AUC value of the ROC curve of Model 1 and Model 2 is less than 0.7, indicating that the quality of the two models is moderate.

**Table 4:** Evaluation indicators for logistic regression models.

Model	Accuracy	Recall	Precision	Cutoff Value	Sensitivity	Specificity	Youden's index	F	AUC (95%CI)
Model 1	0.567	0.567	0.556	0.561	0.505	0.613	0.118	0.526	0.578 (0.564, 0.592)
Model 2	0.65	0.65	0.648	0.508	0.719	0.565	0.283	0.648	0.688 (0.675, 0.701)
Model 3	0.654	0.654	0.652	0.523	0.710	0.588	0.298	0.65	0.701 (0.688, 0.714)

For Model 3, the *P* value of Ominbus test was less than 0.001, the logarithmic likelihood value of −2 was 7994.346, and the *P* value of Hosmer-Lameshaw test was 0.570, greater than 0.05, indicating that the model was of good quality. Model 3 shows that gender, chronic disease status, family debt, whether epidemic control policies have a great impact on respondents’ own lives, self-rated quality of life, perceived pressure, family communication, perceived social support, and self-efficacy are significantly correlated with the risk of subthreshold depression (*P* < 0.05), as shown in Table 5 for details.

**Table 5:** Binary stepwise logistic regression results of factors associated with subthreshold depression among Chinese university students.

Model 1							
Variable	β	SE	Wald *χ2*	*P*	OR	95%CI(Lower)	95%CI(Upper)
Gender (The control group is male)							
Female	0.225	0.054	17.625	＜**0.001**	1.252	1.127	1.390
Major (The control group is social science)							
Non-social science	−0.127	0.053	5.733	**0.017**	0.881	0.794	0.977
Whether suffered from Chronic diseases (The control group is No)							
Yes	0.540	0.097	31.093	＜**0.001**	1.715	1.419	2.074
Whether is an only child (The control group is No)							
Yes	−0.143	0.053	7.370	**0.007**	0.867	0.782	0.961
Whether the respondent's household is in debt (The control group is No)							
Yes	0.262	0.052	25.430	＜**0.001**	1.299	1.173	1.438
Whether the epidemic prevention and control policy have a great impact on respondents (The control group is No)							
Yes	0.168	0.051	10.955	**0.001**	1.183	1.071	1.307
Model 2							
Self-assessment of quality of life (The control group is low score group)							
High score group	−0.505	0.055	84.580	＜**0.001**	0.603	0.542	0.672
Perceived pressure (The control group is low score group)							
High score group	0.533	0.055	92.622	＜**0.001**	1.705	1.529	1.901
Family Communication (The control group is low score group)							
High score group	−0.419	0.056	55.626	＜**0.001**	0.658	0.589	0.734
Perceived Social Support (The control group is low score group)							
High score group	−0.480	0.058	67.721	＜**0.001**	0.619	0.552	0.694
Self efficacy (The control group is low score group)							
High score group	−0.609	0.083	53.185	＜**0.001**	0.544	0.462	0.641
Model 3							
Gender (The control group is male)							
Female	0.306	0.055	30.646	＜**0.001**	1.358	1.219	1.513
Whether suffered from Chronic diseases (The control group is No)							
Yes	0.440	0.102	18.594	＜**0.001**	1.552	1.271	1.895
Whether the respondent's household is in debt (The control group is No)							
Yes	0.227	0.055	17.319	＜**0.001**	1.255	1.128	1.397
Whether the epidemic prevention and control policy have a great impact on respondents (The control group is No)							
Yes	0.281	0.054	27.187	＜**0.001**	1.325	1.192	1.473
Self-assessment of quality of life (The control group is low score group)							
High score group	−0.517	0.056	86.423	＜**0.001**	0.597	0.535	0.665
Perceived pressure (The control group is low score group)							
High score group	0.553	0.056	97.612	＜**0.001**	1.739	1.558	1.940
Family Communication (The control group is low score group)							
High score group	−0.372	0.057	42.881	＜**0.001**	0.689	0.617	0.771
Perceived Social Support (The control group is low score group)							
High score group	−0.512	0.059	75.058	＜**0.001**	0.599	0.534	0.673
Self efficacy (The control group is low score group)							
High score group	−0.597	0.085	49.901	＜**0.001**	0.550	0.466	0.649

In terms of general characteristics, female college students were more likely to have subthreshold depression than male (OR = 1.358, 95%CI 1.219–1.513, *P* < 0.001). Patients with chronic diseases were more likely to have subthreshold depression than those without chronic diseases (OR = 1.552, 95%CI 1.271–1.895, *P* < 0.001). People with household debt were more likely to have subthreshold depression than those without household debt (OR = 1.255, 95%CI 1.128–1.397, *P* < 0.001). People whose lives were more affected by epidemic prevention and control policies were more likely to suffer from subthreshold depression than those whose lives were less affected (OR = 1.325, 95%CI 1.192–1.473, *P* < 0.001).

In terms of scale scores, compared with low quality of life group, high quality of life group was less likely to have subthreshold depression (OR = 0.597, 95%CI 0.535–0.665, *P* < 0.001). Compared with low perceived stress group, high perceived stress group was more likely to have subthreshold depression (OR = 1.739, 95%CI 1.558–1.940, *P* < 0.001). Compared with low family communication group, high family communication group was less likely to have subthreshold depression (OR = 0.689, 95%CI 0.617–0.771, *P* < 0.001). Compared with those with low perceived social support, those with high perceived social support were less likely to have subthreshold depression (OR = 0.599, 95 %10.13039/501100022346CI 0.534–0.673, *P* < 0.001). People in the high self-efficacy group were less likely to have subthreshold depression than those in the low self-efficacy group (OR = 0.550, 95%CI 0.466–0.649, *P* < 0.001).

The selected variables of Model 1 and Model 2 are basically consistent with those of Model 3, which further proves the robustness of Model 3. However, in Model 1, which adopts general characteristics as independent variables, the two variables of “whether I am the only child" and “major" are also screened out. Model 1 showed that the respondents who are the only-child of their family were less likely to have subthreshold depression than non-only child (OR = 0.867, 95%CI 0.782–0.961, *P* = 0.007); Compared with those who majored in social sciences, those who majored in non-social sciences were less likely to have subthreshold depression (OR = 0.881, 95%CI 0.794 to 0.977, *P* = 0.017), which is worthy of further study.

### 7.7 Subgroup analysis results

Subgroups were stratified by gender, major and location, and binary stepwise logistic regression was performed to generate 7 models (see Supplementary Table 2 for models).

In terms of gender, the associated factors of subthreshold depression in male college students included chronic disease status, family debt, whether epidemic control policies had a great impact on normal life, self-rated quality of life, perceived pressure, family communication, perceived social support and self-efficacy (*P* < 0.05). Compared to the model for male students, the model for female students has an additional factor of major (*P* < 0.05).

In terms of majors, the associated factors of subthreshold depression among social science students included gender, family debt, whether epidemic control policies had a great impact on their own life, self-rated quality of life, perceived pressure, family communication, perceived social support, and self-efficacy (*P* < 0.05). Factors related to the risk of subthreshold depression in non-social science college students included gender, Whether suffering from chronic diseases, location, whether they are the only child, whether the epidemic control policy had a great impact on their own life, self-rated quality of life, perceived pressure, family communication, perceived social support, and self-efficacy (*P* < 0.05).

In terms of location, the associated factors of subthreshold depression of college students in western China included whether they have chronic disease status, whether there are family debts, whether the epidemic control policies had a great impact on their own life, self-rated quality of life, perceived pressure, family communication, perceived social support, and self-efficacy (*P* < 0.05). Factors related to the risk of subthreshold depression of college students in central China included gender, whether there are family debts, whether the epidemic control policy had a great impact on their own life, self-rated quality of life, perceived pressure, family communication, perceived social support, and self-efficacy (*P* < 0.05). The associated factors of subthreshold depression of college students in eastern China included gender, major, whether they have chronic disease status, whether the epidemic control policies had a great impact on their own life, self-rated quality of life, perceived pressure, family communication, perceived social support, and self-efficacy (*P* < 0.05).

## 8 Discussion

### 8.1 The current status of subthreshold depression among Chinese college students

This study assessed the prevalence of subthreshold depression among Chinese college students using the PHQ-9. The findings suggest that subthreshold depression is more prevalent (39.7 %) than depression (28.4 %) among respondents. Additionally, subthreshold depression accounts for 55.5 % of all non-depression respondents. Subthreshold depression and depression are currently the main psychological problems among Chinese college students. More than half of Chinese college students may have tendencies towards depression or suffer from depression. Subthreshold depression may be more common among college students than depression. This study's findings share similarities and differences with related research. In 2018, Dalky and Gharaibeh et al. surveyed 600 Jordanian university students and discovered that 54.4 % of participants experienced mental health issues, including varying degrees of subthreshold depression symptoms such as anxiety and stress. Surveys conducted in countries such as Malaysia have yielded similar results, with depression tendencies, anxiety, and high levels of stress among college students prevalent and on the rise [42]. Lee et al.'s (2020) survey of 2691 American college students revealed that over one-third experienced varying degrees of anxiety and depression during the COVID-19 pandemic [43]. Similarly, a 2015 study by the American Association for Anxiety and Depression revealed that college students are more likely to experience depressive symptoms than American adults, with over 70 % reporting symptoms of depression [44]. These findings highlight the global issue of subthreshold depression as a prodromal symptom of depression. Therefore, it is crucial to prioritize the psychological well-being of college students in different countries and provide them with necessary support. In 2020, Langer et al. conducted a study in Chile which revealed that during the COVID-19 pandemic, 14.3 % of college students experienced subthreshold depression, while 32.2 % met the criteria for major depressive disorder (MDD) [12]. The higher prevalence of MDD may be attributed to the pandemic's effects on mental health. The pandemic has increased the risk for depression, anxiety, and insomnia, which may exacerbate subthreshold depression [45].The study found correlations between depressive symptoms and Big Five personality traits, specifically neuroticism, extraversion, and conscientiousness. These results suggest that individuals with subthreshold depression may have different personality traits compared to those without depressive symptoms. Therefore, as the personality moderates the relationship between COVID-19 and depression level, COVID-19 exacerbates the differentiation between individuals with severe depression and those with minimal depressive symptoms [46], leading to a higher prevalence of MDD among Chilean university students than subthreshold depression during the pandemic.

Previous research has examined the occurrence of subthreshold depression in adults in China. According to Liao et al. (2022), the prevalence of subthreshold depression in adults in primary health care ranged from 2.9 % to 9.9 %, while community studies reported rates of 1.4 %–17.2 % [47]. The study carried out by Liao et al. in Guangdong Province, China in 2021 revealed that the prevalence of subthreshold depression in adults was 14.7 % [48], which is lower than the rate found among college students in this study. This indicates that college students are a high-risk group for subthreshold depression due to the academic, financial [49], and social pressures they face, particularly during the critical period of personality formation and development. The COVID-19 pandemic has increased the psychological burden by causing more isolation and avoidance behaviours, which may lead to subthreshold depression [50].

Research data shows that the prevalence of subthreshold depression is high among both males (51.3 %) and females (58.2 %), indicating similarities and differences with previous studies. Langer and others, in their 2020 study, revealed that the prevalence of subthreshold depression among university students of different countries was only 13.6 % for males and 14.7 % for females, much lower than the results of this study [51]. In their 2012 study on subthreshold depression among Chinese university students, Yang et al. found that moderate depression is a common occurrence [52]. This indicates that the severity of subthreshold depression in Chinese university students is relatively high compared to global standards. The higher rates of subthreshold depression among different gender groups of Chinese university students could be due to negative peer effects resulting from intense competition [53]. As mentioned above, the high prevalence of subthreshold depression among Chinese college students may also be related to the COVID-19 epidemic.

### 8.2 Differences between Chinese college students with and without subthreshold depression

This study compared depression symptoms between respondents with and without subthreshold depression using t-tests. The results showed significant differences between the two groups in their perception of self-favorability, family attitude, and attention concentration, consistent with previous research. Research indicates that symptoms of depression can lead to negative biases in personal memory and attention, resulting in negative emotions and exacerbating the impact of adverse events on one's life [54]. Thus, individuals with subthreshold depression are more likely to focus on negative aspects, resulting in negative perceptions of themselves and those around them compared to those without subthreshold depression symptoms. Depression is often associated with self-criticism, reduced self-esteem, and greater dependence on others [55]. In a related study on post-traumatic stress disorder (PTSD) and the combined network of depression symptoms, Duek et al. found that lack of attention is a core feature of the combined network of depression and PTSD [56].

It is important to note that this study found only minor differences between patients with subthreshold depression and those without, in terms of behaviour, speech patterns, and thoughts of suicide or self-harm. This contrasts with previous research. In a 2019 survey of Hong Kong residents, Hou et al. (2019) reported that around 25.7 % of people experienced depressive symptoms, which was significantly higher than the proportion of individuals with suicidal ideation (9.1 %) [57]. A study conducted by Gijzen et al., in 2019 also demonstrated that adolescents with higher depression symptom scores were more likely to report suicidal ideation [58]. The discrepancy in suicide rates may be due to the multifaceted nature of this social phenomenon, which is influenced by various factors such as employment and economic pressure. These factors may be temporary and not necessarily accompanied by other depressive symptoms [59].

### 8.3 Related factors of subthreshold depression risk among Chinese college students

This study employed multivariate binary logistic regression to investigate the general characteristics of the respondents and the correlation between the scoring of certain scales and the risk of subthreshold depression. Female college students, individuals with chronic diseases, those with family debt, and those whose lives are greatly affected by epidemic control policies are at a higher risk of developing subthreshold depression. Conversely, individuals with high self-evaluation of their quality of life, low perceived stress, high family communication, a good understanding of social support, and high self-efficacy are at a lower risk of developing subthreshold depression.These findings are in line with previous research. Compared to men, women may be more likely to experience mental health problems due to their tendency towards greater emotional intensity and sensitivity [60]. Research has indicated that female college students are more prone to developing depression than their male counterparts. This is attributed to academic performance, physical appearance, and lifestyle habits, as well as the pressure from school and life [61]. Research indicates that female college students are more susceptible to subthreshold depression than their male counterparts. Existing research shows that there is a high correlation between chronic diseases and psychological diseases such as depression [62]. Moussavi et al. investigated the correlation between chronic diseases, including angina, arthritis, asthma, and diabetes, and mental health using data from the World Health Survey. The findings indicate that individuals with one or more chronic diseases are at a greater risk of experiencing depression and other related psychological disorders than those without such conditions. Research has shown that the financial burden of family debt is related to the risk of subthreshold depression. Family debt can exacerbate financial difficulties. College students who do not have family debt are less likely to experience financial stress and are therefore less susceptible to subthreshold depression compared to those who have debt [63]. Moreover, the COVID-19 pandemic and the consequent measures taken to control the epidemic have had a significant impact on people's mental health, including subthreshold depression [64]. This study's scale score grading results are consistent with previous research conclusions. Büsselmann et al. (2018) found that a higher quality of life among German respondents can alleviate symptoms of depression and reduce the severity of emotions such as despair [65].The study found that individuals with a high self-evaluation of their quality of life were less likely to experience subthreshold depression when controlling for other relevant variables. This may be due to the close relationship between quality of life and psychological distress factors. Individuals with higher self-rated quality of life may be less affected by negative emotions, resulting in a lower risk of subthreshold depression [66]. Secondly, Gao et al.'s study in 2020 in China showed that stress, especially peer pressure, increased the tendency of college students to experience depression [67].This study found a significant positive correlation between perceived stress and the risk of subthreshold depression. This is because individuals experiencing excessive psychological pressure are more likely to experience negative emotions, which can lead to depressive symptoms [68]. 10.13039/100014337Furthermore, a systematic review of middle-aged and elderly individuals in Asian countries has shown that strong social support can alleviate depressive symptoms. Notably, family support and communication have been found to be particularly effective in promoting healthy attitudes in Asian countries when compared to Western countries [69].Research has shown that college students and the general adult population share similar psychological states. Additionally, high self-efficacy has been found to boost individuals' confidence, increase their perception of certainty about themselves and their social environment, and reduce their pressure to face uncertainty and the associated risks of subthreshold depression [70].

It should be noted that, in comparison to Model 3, Model 1 suggests that being an only child and the choice of major may also be linked to the risk of subthreshold depression. Specifically, non-only children and students majoring in social sciences exhibit a higher risk of developing subthreshold depression. Additionally, previous studies have provided evidence that supports the conclusions made in this study regarding the variable of major. On one hand, exposure to cultural diversity may make social science students more aware of the unequal distribution of social resources, which can lead to political disillusionment [71]. On the other hand, individuals who possess greater knowledge of social and political affairs are less likely to alter their inherent biases. This can result in the development of hostile emotions towards those with differing opinions, leading to increased symptoms of anxiety, stress, and depression [72].

Such negative emotions can harm mental health, especially when coupled with different political party identities in American politics [73].

In addition, social science students may be more concerned about their future employment prospects and thus more likely to develop depressive tendencies compared to non-social science students. In terms of the only child variable, there are both similarities and differences between the results of this study and previous studies. Liu et al. conducted research in China in 2020 and found that being an only child was not related to anxiety levels [74]. However, previous studies conducted during the early stages of the one-child policy in China have come to the same conclusion as this study, that is, compared with only children, non-only children tend to have more psychological problems such as depression [75]. This difference may be related to the age of the sample and their ranking among siblings. Research shows that among non-only children, respondents who are not the eldest are less likely to suffer from depression and other mental illnesses [76]. With the adjustments made to China's family planning policy, the implementation of the single two-child, comprehensive two-child and comprehensive three-child policies has meant that the majority of the student respondents in this study are likely to be the eldest siblings in their families. This may mean that they receive more attention and care from their parents, which could have a positive impact on their psychological status. Research has shown that a comprehensive two-child policy may lead to more internal conflicts within families, which are mainly reflected in the negotiation process of decision-making in family matters. Therefore, more conflict may make respondents more vulnerable to mental health problems [77]. In addition, only children may have a higher tolerance for loneliness caused by isolation, while non-only children are more likely to experience psychological distress caused by isolation.

The comparison results between Model 1 and Model 3 indicate that after the inclusion of the self-rating scale score grading variable, the correlation between major and being an only child on the risk of subthreshold depression among Chinese college students is no longer significant. This indicates that the effects of two variables, namely college students' major and whether they are only children, on the risk of subthreshold depression are not stable, and further research is needed to investigate the complex relationship between these factors.

### 8.4 Differences in related factors of subthreshold depression risk among different subgroups of the population

In this study, subgroups were classified based on gender, major and location of respondents to test for differences in relevant factors of subthreshold depression risk between different populations. The results show that major is associated with the risk of subthreshold depression in female students, but not in male students. This is in line with existing research. On the one hand, female students may be more vulnerable to academic influences. On the other hand, female students may be more sensitive to perspective factors. So the prospect of their major is more likely to have an impact on the risk of subthreshold depression in female students [78]. Second, the study found that chronic illness status and single-child status were significant factors influencing the risk of subthreshold depression among non-social science students, but not among social science students. (*P* < 0.05). These findings suggest that the psychological status of social science students is more influenced by socio-economic factors and less by personal physical factors, innate dispositions or the immediate environment. This may be related to the content of their major. Social sciences focus on social knowledge, such as social stratification and social justice, so social science students are less sensitive to non-structural factors such as personal health [79]. Third, there are different factors associated with the risk of subthreshold depression among college students in western, central and eastern China. Among them, the relevant factors for the risk of subthreshold depression among college students in western China included permanent residence, chronic disease prevalence, family debt situation, and whether epidemic control policies have a significant impact on their own lives (*P* < 0.05); the relevant factors for the risk of subthreshold depression among college students in central China also included gender (*P* < 0.05) The relevant factors for the risk of subthreshold depression among college students in East China included gender (*P* < 0.05) and major (*P* < 0.05), but not permanent residence and family debt situation. The difference in the factors associated with subthreshold depression in different regions reflects the different impact of family structure, economic development and social environment on individual psychological status in different regions. Research has shown that events in different economic and social environments can interfere with an individual's efforts to achieve life goals, thereby affecting their psychological state [80].

### 8.5 Suggestions

Firstly, since the occurrence of subthreshold depression is influenced by the socio-economic environment of different regions, the government should take measures to improve the level of regional economic development and narrow the gap between different regions. This could help to increase students' sense of economic security and reduce the negative impact of financial pressures on their mental health. On the other hand, the government is obliged to mobilise experts, scientists and social organisations to participate in mental health construction projects and to provide treatment support for people with subthreshold depression. Secondly, there is a need for a collaborative governance mechanism involving the state, communities, schools and families to address the issue of subthreshold depression among college students by strengthening support for their social networks, including advocating for the establishment of good family communication relationships, To promote communication among college students from different majors to build a social support network and reduce the risk of subthreshold depression, colleges can conduct various forms of mental health education activities, such as setting up psychological hotlines, organizing mental health knowledge competitions, conducting sports check-in activities, and providing group psychological counseling to popularize basic mental health knowledge. Thus, students could establish mental health awareness, understand certain psychological adjustment methods and recognise psychological abnormalities. Thirdly, it is crucial for college teachers not only to guide students in developing a positive sense of self-efficacy, but also to focus their attention on the mental health concerns of female students and those facing financial difficulties. By providing targeted support and resources, teachers can help students develop healthy coping strategies to effectively manage the academic and peer pressures they face.

### 8.6 Research advantages and limitations

The advantage of this study lies in the use of scientifically sound sample selection methods and research tools to conduct a large-scale sample analysis of nearly 10,000 college students, undergraduates and graduate students from different regions, majors and educational backgrounds in China. The diverse sample allows for an effective representation of the current state of subthreshold depression among Chinese college students, as well as the identification of relevant risk factors. At the same time, this study implemented robustness tests through subgroup analysis and other methods. A total of seven subgroup analysis models were constructed, highlighting the similarities and differences of factors contributing to subthreshold depression among college students of different genders, majors, and regions, which not only enriched the content of the study, but also provided an empirical basis for implementing targeted interventions.

This study has some limitations that should be acknowledged. First, it relies on a self-report scale to measure the variables, which may lead to recall bias. Secondly, this study did not conduct specific empirical analyses and tests of the mechanisms by which each variable affects the risk of SD. Therefore, future research can focus on the causes and mechanisms of the effects of each variable on the risk of subthreshold depression, with particular attention to the main and only child status variables. Thirdly, MDD, also known as clinical depression, is a mental disorder characterized by at least two weeks of pervasive low mood, low self-esteem, and loss of interest or pleasure in normally enjoyable activities [80]. The clinical diagnosis of MDD requires professional assessment. The PHQ-9 scale used in this study only measure depressive symptoms, which may be subject to inaccuracies or confounding factors. The reported prevalence of MDD in this paper may differ slightly from the actual prevalence. Finally, this study is cross-sectional and cannot determine the causal relationship between the occurrence of subthreshold depression and other variables. Therefore, future research can focus on long-term panel data research and analysis and causal mechanism testing.

## 9 Conclusion

The main findings of this study can be summarised as follows. Firstly, the prevalence rate of subthreshold depression among students is higher than that of depression. The proportion of students with subthreshold depression is almost 40 % of all students. Second, this study identified factors that may be associated with the risk of subthreshold depression among Chinese college students, including demographic characteristics and basic family information, as well as self-rated quality of life, perceived stress, family communication status, perceived social support, and self-efficacy. Results showed that being female, having poor physical health, being affected by epidemic policies, having high perceived stress, and having family debt were risk factors for the prevalence of subthreshold depression among Chinese college students. On the other hand, high family communication, high understanding of social support and high self-efficacy may be protective factors against subthreshold depression. This study suggests the mobilisation and integration of resources from various entities, including the country, community, university and family, to develop programmes to improve the mental health of Chinese college students.

## Funding

This study was supported by the University of Macau Foundation (MYRG106 (Y1- L3)-ICMS13-BY), University of Macau Foundation (MYRG2015-00190-ICMSQRCM), University of Macau Foundation (MYRG2022-00257-ICMS) and University of Macau Foundation (SKL-QRCM-IRG2023-032).

## Ethics Statement

This study was approved by the Ethics Research Committee of the Health Culture Research Center of Shaanxi (No. JKWH-2022-02). All research procedures were conducted by the principles outlined in the Declaration of Helsinki and the guidelines provided by the relevant regulatory bodies. The research was conducted with the voluntary participation of human subjects who provided written informed consent before being included in the study. Participant confidentiality and anonymity were preserved throughout the data collection, analysis, and reporting processes. Measures were taken to ensure that no harm came to any participants as a result of their involvement in the study. Any potential risks or adverse effects of the research were carefully considered and managed to minimize harm. Data was stored securely and accessed only by authorized personnel for purposes related to the research project. Results have been reported in aggregate form and individual participants cannot be identified from the presented data. If you have any concerns about the research practices employed in this study, please contact Pu Ge, at email 17853140673@163.com.

## Data availability statement

The original data of the study can be obtained from the corresponding author of this paper, Dr Yibo Wu (E-mail:bjmuwuyibo@outlook.com), upon reasonable request.

## CRediT authorship contribution statement

**Pu Ge:** Writing – review & editing, Writing – original draft, Investigation, Formal analysis, Data curation, Conceptualization. **Cheng Tan:** Writing – review & editing, Writing – original draft. **Jia-xin Liu:** Writing – review & editing, Writing – original draft. **Qiong Cai:** Writing – review & editing. **Si-qi Zhao:** Writing – review & editing. **Wen-ying Hong:** Writing – review & editing. **Kun-meng Liu:** Writing – review & editing. **Jia-le Qi:** Writing – review & editing. **Chen Hu:** Writing – review & editing. **Wen-li Yu:** Writing – review & editing. **Yi-miao Li:** Writing – review & editing. **Yuan You:** Writing – review & editing. **Jin-han Guo:** Writing – review & editing. **Ming-yan Hao:** Writing – review & editing. **Yang Chen:** Writing – review & editing. **Lu-tong Pan:** Writing – review & editing. **Di-yue Liu:** Writing – review & editing. **Meng-yao Yan:** Writing – review & editing. **Jin-zi Zhang:** Writing – review & editing. **Qi-yu Li:** Writing – review & editing. **Bo-ya Sun:** Writing – review & editing. **Xiao Han:** Writing – review & editing. **Fuer Mo:** Writing – review & editing. **Ying Bian:** Writing – review & editing, Writing – original draft, Supervision, Funding acquisition, Conceptualization, Wu, Writing – review & editing, Supervision, Investigation, Conceptualization.

## Declaration of competing interest

The authors declare that they have no known competing financial interests or personal relationships that could have appeared to influence the work reported in this paper.
