# Supplementary material for: Prevalence of subthreshold depression and its related factors in Chinese college students: A cross-sectional study
Source: Heliyon. 2024 Jun 6;10(12):e32595. doi: 10.1016/j.heliyon.2024.e32595 (PMC11233893; doi:10.1016/j.heliyon.2024.e32595)
Supplement: Multimedia component 1 [file mmc1.docx]

[Supplementary](javascript:;) Table 1:Quality control of PBICR-2022

| **Procedure** | **Measures** |
| --- | --- |
| **Questionnaire design** | Researchers initially designed the questionnaire by reading the related literature scientifically and comprehensively. Before the questionnaire was formally used, 38 experts with senior titles in the subject areas of social medicine, behavioral epidemiology etc. were invited to meetings from March to June 2022 to discuss the content of the questionnaire. At the same time, the research group invited experts in psychology, health education, health statistics, Health service management, humanistic medicine, journalism and communication, clinical medicine, pharmacy, nursing, sociology, philosophy and other disciplines to carefully review the content of the questionnaire and propose Modification suggestions to it. The members of PBICR-2022 carefully revised the questionnaire according to the experts' Suggestions. |
| **Pre-investigation stage** | The researchers conducted three rounds of pilot study from June 5 to June 8, June 10 to June 13, and June 15 to June 18, 2022. The sampling method of the pilot study was quota sampling with the same quota proportion as the official investigation. The sample sizes of the three surveys were 100, 100 and 200, respectively. In the pilot study, members of the research team collected and organized the feedback from the respondents, assessed the reliability and validity of the questionnaire through statistical analysis, and revised and resubmitted the questionnaire to the experts for review based on the feedback from the respondents and the results of the reliability and validity after the discussion among the members of the research team. After three rounds of pilot study, the questionnaire used for the formal investigation was finalized. The questionnaires collected in the pilot study were not included in the final statistical analysis. |
| **Investigators' training** | The research team posted an online announcement for the recruitment of provincial leaders and investigators, both of whom were required to be students in universities. The research group screened preliminarily. based on the resumes and conducted information verification and communication skills tests for those who passed the resume screening. The research team conducted five unified training sessions for potential investigators on May 26, May 28, May 31, June 3, and June 10, 2022, with each session lasting about 1 hour and 30 minutes, during which the trainers answered the trainees' questions. After the five training sessions, the research team assessed the trainees and assigned tasks to the trainees who passed the test. |
| **Questionnaire distribution** | During the questionnaire distribution, the investigators followed the principles of scientific research design in order to minimize bias in the study. The investigators registered and coded the completed questionnaires. Before starting the formal investigation, the researcher re-emphasized the precautions to the investigators to ensure the pass rate of the Collected questionnaires. Every Sunday evening, the researcher communicated with the head of investigators in each provincial administrative district to summarize and evaluate the collected questionnaires, promptly point out problems in the survey, supervise the work of the head of the provincial investigators, and discuss, identify, and provide guidance on issues of disagreement in the investigation in real time. |
| **Questionnaire screening** | According to the established questionnaire screening criteria, two research team members logically checked and screened the questionnaires independently to exclude the unqualified ones. |

[Supplementary](javascript:;) Table 2:Subgroup analysis of the risk of subthreshold depression by binary step-up logistic regression.

| Model 1 Male | | | | | | | | |
| --- | --- | --- | --- | --- | --- | --- | --- | --- |
| Variable | β | SE | Wald χ2 | | *P* | OR | 95%CI  (Lower) | 95%CI  (Upper) |
| Whether suffered from Chronic diseases  (The control group is No) |  |  |  | |  |  |  |  |
| Yes | 0.594 | 0.161 | 13.678 | | ＜0.001 | 1.811 | 1.322 | 2.481 |
| Whether the respondent's household is in debt  (The control group is No) |  |  |  | |  |  |  |  |
| Yes | 0.268 | 0.087 | 9.514 | | 0.002 | 1.308 | 1.103 | 1.551 |
| Whether the epidemic prevention and control policy have a great impact on respondents(The control group is No) |  |  |  | |  |  |  |  |
| Yes | 0.206 | 0.085 | 5.840 | | 0.016 | 1.229 | 1.040 | 1.453 |
| Self-assessment of quality of life  (The control group is low score group) |  |  |  | |  |  |  |  |
| High score group | -0.467 | 0.088 | 27.947 | | ＜0.001 | 0.627 | 0.527 | 0.745 |
| Perceived pressure  (The control group is low score group) |  |  |  | |  |  |  |  |
| High score group | 0.430 | 0.088 | 23.947 | | ＜0.001 | 1.538 | 1.294 | 1.827 |
| Family Communication  (The control group is low score group) |  |  |  | |  |  |  |  |
| High score group | -0.322 | 0.090 | 12.706 | | ＜0.001 | 0.725 | 0.607 | 0.865 |
| Perceived Social Support  (The control group is low score group) |  |  |  | |  |  |  |  |
| High score group | -0.586 | 0.095 | 38.080 | | ＜0.001 | 0.557 | 0.462 | 0.671 |
| Self efficacy(The control group is low score group) |  |  |  | |  |  |  |  |
| High score group | -0.468 | 0.127 | 13.534 | | ＜0.001 | 0.626 | 0.488 | 0.804 |
| Model 2 Female | | | | | | | | |
| Major(The control group is social science) |  |  |  | |  |  |  |  |
| Non-social science | -0.148 | 0.069 | 4.546 | | 0.033 | 0.862 | 0.753 | 0.988 |
| Whether suffered from Chronic diseases  (The control group is No) |  |  |  | |  |  |  |  |
| Yes | 0.335 | 0.132 | 6.427 | | 0.011 | 1.398 | 1.079 | 1.811 |
| Whether the respondent's household is in debt  (The control group is No) |  |  |  | |  |  |  |  |
| Yes | 0.197 | 0.070 | 7.786 | | 0.005 | 1.217 | 1.060 | 1.397 |
| Whether the epidemic prevention and control policy have a great impact on respondents(The control group is No) |  |  |  | |  |  |  |  |
| Yes | 0.325 | 0.070 | 21.604 | | ＜0.001 | 1.384 | 1.207 | 1.588 |
| Self-assessment of quality of life  (The control group is low score group) |  |  |  | |  |  |  |  |
| High score group | -0.555 | 0.072 | 59.697 | | ＜0.001 | 0.574 | 0.499 | 0.661 |
| Perceived pressure  (The control group is low score group) |  |  |  | |  |  |  |  |
| High score group | 0.645 | 0.073 | 78.047 | | ＜0.001 | 1.906 | 1.652 | 2.200 |
| Family Communication  (The control group is low score group) |  |  |  | |  |  |  |  |
| High score group | -0.405 | 0.073 | 30.326 | | ＜0.001 | 0.667 | 0.578 | 0.771 |
| Perceived Social Support  (The control group is low score group) |  |  |  | |  |  |  |  |
| High score group | -0.464 | 0.076 | 37.388 | | ＜0.001 | 0.629 | 0.542 | 0.730 |
| Self efficacy(The control group is low score group) |  |  |  | |  |  |  |  |
| High score group | -0.703 | 0.114 | 38.140 | | ＜0.001 | 0.495 | 0.396 | 0.619 |
| Model 3 Social Science | | | | | | | | |
| Gender(The control group is male) |  |  |  | |  |  |  |  |
| Female | 0.391 | 0.096 | 16.603 | | ＜0.001 | 1.478 | 1.225 | 1.783 |
| Place of permanent residence(The control group is rural) |  |  |  | |  |  |  |  |
| Urban | 0.204 | 0.101 | 4.030 | | 0.045 | 1.226 | 1.005 | 1.495 |
| Whether the respondent's household is in debt  (The control group is No) |  |  |  | |  |  |  |  |
| Yes | 0.306 | 0.085 | | 12.785 | ＜0.001 | 1.358 | 1.148 | 1.605 |
| Whether the epidemic prevention and control policy have a great impact on respondents(The control group is No) |  |  |  | |  |  |  |  |
| Yes | 0.335 | 0.085 | 15.547 | | ＜0.001 | 1.398 | 1.184 | 1.652 |
| Self-assessment of quality of life  (The control group is low score group) |  |  |  | |  |  |  |  |
| High score group | -0.699 | 0.087 | 64.417 | | ＜0.001 | 0.497 | 0.419 | .590 |
| Perceived pressure(The control group is low score group) |  |  |  | |  |  |  |  |
| High score group | 0.647 | 0.089 | 53.010 | | ＜0.001 | 1.911 | 1.605 | 2.274 |
| Family Communication  (The control group is low score group) |  |  |  | |  |  |  |  |
| High score group | -0.398 | 0.089 | 19.984 | | ＜0.001 | 0.672 | 0.564 | 0.800 |
| Perceived Social Support  (The control group is low score group) |  |  |  | |  |  |  |  |
| High score group | -0.465 | 0.093 | 25.165 | | ＜0.001 | 0.628 | 0.524 | 0.753 |
| Self efficacy(The control group is low score group) |  |  |  | |  |  |  |  |
| High score group | -0.476 | 0.131 | 13.276 | | ＜0.001 | 0.621 | 0.481 | 0.802 |
| Model 4 Non-social Science | | | | | | | | |
| Gender(The control group is male) |  |  |  | |  |  |  |  |
| Female | 0.210 | 0.071 | 8.810 | | 0.003 | 1.234 | 1.074 | 1.418 |
| Whether suffered from Chronic diseases  (The control group is No) |  |  |  | |  |  |  |  |
| Yes | 0.582 | 0.134 | 18.861 | | ＜0.001 | 1.789 | 1.376 | 2.326 |
| Whether is an only child(The control group is No) |  |  |  | |  |  |  |  |
| Yes | -0.186 | 0.072 | 6.629 | | 0.010 | 0.830 | 0.721 | 0.957 |
| Whether the respondent's household is in debt  (The control group is No) |  |  |  | |  |  |  |  |
| Yes | 0.165 | 0.072 | 5.328 | | 0.021 | 1.180 | 1.025 | 1.358 |
| Whether the epidemic prevention and control policy have a great impact on respondents(The control group is No) |  |  |  | |  |  |  |  |
| Yes | 0.234 | 0.070 | 11.093 | | 0.001 | 1.264 | 1.101 | 1.451 |
| Self-assessment of quality of life  (The control group is low score group) |  |  |  | |  |  |  |  |
| High score group | -0.397 | 0.073 | 29.924 | | ＜0.001 | 0.672 | 0.583 | 0.775 |
| Perceived pressure(The control group is low score group) |  |  |  | |  |  |  |  |
| High score group | 0.498 | 0.073 | 46.982 | | ＜0.001 | 1.645 | 1.427 | 1.896 |
| Family Communication(The control group is low score group) |  |  |  | |  |  |  |  |
| High score group | -0.343 | 0.074 | 21.390 | | ＜0.001 | 0.710 | 0.614 | 0.821 |
| Perceived Social Support  (The control group is low score group) |  |  |  | |  |  |  |  |
| High score group | -0.549 | 0.077 | 50.333 | | ＜0.001 | 0.578 | 0.496 | 0.672 |
| Self efficacy(The control group is low score group) |  |  |  | |  |  |  |  |
| High score group | -0.687 | 0.112 | 37.818 | | ＜0.001 | 0.503 | 0.404 | 0.626 |
| Model 5 Western China | | | | | | | | |
| Whether suffered from Chronic diseases  (The control group is No) |  |  |  | |  |  |  |  |
| Yes | 0.376 | 0.167 | 5.046 | | 0.025 | 1.456 | 1.049 | 2.022 |
| Place of permanent residence(The control group is rural) |  |  |  | |  |  |  |  |
| Urban | 0.232 | 0.114 | 4.158 | | 0.041 | 1.262 | 1.009 | 1.577 |
| Whether the respondent's household is in debt  (The control group is No) |  |  |  | |  |  |  |  |
| Yes | 0.340 | 0.097 | 12.394 | | ＜0.001 | 1.405 | 1.163 | 1.698 |
| Whether the epidemic prevention and control policy have a great impact on respondents(The control group is No) |  |  |  | |  |  |  |  |
| Yes | 0.309 | 0.097 | 10.126 | | 0.001 | 1.362 | 1.126 | 1.647 |
| Self-assessment of quality of life  (The control group is low score group) |  |  |  | |  |  |  |  |
| High score group | -0.594 | 0.099 | 35.698 | | ＜0.001 | 0.552 | 0.455 | 0.671 |
| Perceived pressure(The control group is low score group) |  |  |  | |  |  |  |  |
| High score group | 0.679 | 0.100 | 45.849 | | ＜0.001 | 1.971 | 1.620 | 2.399 |
| Family Communication(The control group is low score group) |  |  |  | |  |  |  |  |
| High score group | -0.404 | 0.102 | 15.804 | | ＜0.001 | 0.668 | 0.547 | 0.815 |
| Perceived Social Support  (The control group is low score group) |  |  |  | |  |  |  |  |
| High score group | -0.434 | 0.106 | 16.700 | | ＜0.001 | 0.648 | 0.526 | 0.798 |
| Self efficacy(The control group is low score group) |  |  |  | |  |  |  |  |
| High score group | -0.419 | 0.154 | 7.379 | | 0.007 | 0.658 | 0.486 | 0.890 |
| Model 6 Central China | | | | | | | | |
| Gender(The control group is male) |  |  |  | |  |  |  |  |
| Female | 0.390 | 0.108 | 13.060 | | ＜0.001 | 1.476 | 1.195 | 1.824 |
| Whether the respondent's household is in debt  (The control group is No) |  |  |  | |  |  |  |  |
| Yes | 0.318 | 0.107 | 8.745 | | 0.003 | 1.374 | 1.113 | 1.696 |
| Whether the epidemic prevention and control policy have a great impact on respondents(The control group is No) |  |  |  | |  |  |  |  |
| Yes | 0.375 | 0.105 | 12.683 | | ＜0.001 | 1.455 | 1.184 | 1.789 |
| Self-assessment of quality of life  (The control group is low score group) |  |  |  | |  |  |  |  |
| High score group | -0.556 | 0.108 | 26.701 | | ＜0.001 | 0.573 | 0.464 | 0.708 |
| Perceived pressure(The control group is low score group) |  |  |  | |  |  |  |  |
| High score group | 0.550 | 0.109 | 25.555 | | ＜0.001 | 1.733 | 1.400 | 2.145 |
| Family Communication  (The control group is low score group) |  |  |  | |  |  |  |  |
| High score group | -0.342 | 0.109 | 9.760 | | 0.002 | 0.710 | 0.573 | 0.880 |
| Perceived Social Support  (The control group is low score group) |  |  |  | |  |  |  |  |
| High score group | -0.657 | 0.113 | 33.794 | | ＜0.001 | 0.518 | 0.415 | 0.647 |
| Self efficacy(The control group is low score group) |  |  |  | |  |  |  |  |
| High score group | -0.732 | 0.163 | 20.257 | | ＜0.001 | 0.481 | 0.350 | 0.662 |
| Model 7 Eastern China | | | | | | | | |
| Gender(The control group is male) |  |  |  | |  |  |  |  |
| Female | 0.330 | 0.089 | 13.823 | | ＜0.001 | 1.391 | 1.169 | 1.655 |
| Major(The control group is social science) |  |  |  | |  |  |  |  |
| Non-social science | -0.203 | 0.086 | 5.577 | | 0.018 | 0.816 | 0.689 | 0.966 |
| Whether suffered from Chronic diseases  (The control group is No) |  |  |  | |  |  |  |  |
| Yes | 0.483 | 0.155 | 9.682 | | 0.002 | 1.622 | 1.196 | 2.199 |
| Whether the epidemic prevention and control policy have a great impact on respondents(The control group is No) |  |  |  | |  |  |  |  |
| Yes | 0.213 | 0.083 | 6.495 | | 0.011 | 1.237 | 1.050 | 1.457 |
| Self-assessment of quality of life  (The control group is low score group) |  |  |  | |  |  |  |  |
| High score group | -0.429 | 0.086 | 24.743 | | ＜0.001 | 0.651 | 0.550 | 0.771 |
| Perceived pressure(The control group is low score group) |  |  |  | |  |  |  |  |
| High score group | 0.482 | 0.087 | 30.636 | | ＜0.001 | 1.619 | 1.365 | 1.921 |
| Family Communication(The control group is low score group) |  |  |  | |  |  |  |  |
| High score group | -0.368 | 0.089 | 17.250 | | ＜0.001 | 0.692 | 0.582 | 0.823 |
| Perceived Social Support  (The control group is low score group) |  |  |  | |  |  |  |  |
| High score group | -0.492 | 0.093 | 28.279 | | ＜0.001 | 0.611 | 0.510 | 0.733 |
| Self efficacy(The control group is low score group) |  |  |  | |  |  |  |  |
| High score group | -0.649 | 0.130 | 24.756 | | ＜0.001 | 0.523 | 0.405 | 0.675 |
